# Current Challenges in Chronic Bronchial Infection in Patients with Chronic Obstructive Pulmonary Disease

**DOI:** 10.3390/jcm9061639

**Published:** 2020-05-28

**Authors:** José Luis Lopez-Campos, Marc Miravitlles, David de la Rosa Carrillo, Rafael Cantón, Juan Jose Soler-Cataluña, Miguel Angel Martinez-Garcia

**Affiliations:** 1 Unidad Médico-Quirúrgica de Enfermedades Respiratorias, Instituto de Biomedicina de Sevilla (IBiS), Hospital Universitario Virgen del Rocío/Universidad de Sevilla, 41013 Seville, Spain; 2 CIBER de Enfermedades Respiratorias (CIBERES), Instituto de Salud Carlos III, 28029 Madrid, Spain; marcm@separ.es (M.M.); mianmartinezgarcia@gmail.com (M.A.M.-G.); 3 Pneumology Department, Hospital Universitari Vall d’Hebron/Vall d’Hebron Institut de Recerca (VHIR), Vall d’Hebron Barcelona Hospital Campus, 08035 Barcelona, Spain; 4 Pneumology Department, Hospital de la Santa Creu i Sant Pau, 08041 Barcelona, Spain; david.rosa23@gmail.com; 5 Servicio de Microbiología, Hospital Universitario Ramón y Cajal and Instituto Ramón y Cajal de Investigación Sanitaria (IRYCIS), 28034 Madrid, Spain; rafael.canton@salud.madrid.org; 6 Pneumology, Hospital Arnau de Villanova-Lliria, 46015 Valencia, Spain; jjsoler@telefonica.net; 7 Pneumology Department, Universitary and Polytechnic La Fe Hospital, 46015 Valencia, Spain

**Keywords:** COPD, potentially pathogenic microorganism, chronic bronchial infection

## Abstract

Currently, chronic obstructive pulmonary disease (COPD) patients and their physicians face a number of significant clinical challenges, one of which is the high degree of uncertainty related to chronic bronchial infection (CBI). By reviewing the current literature, several challenges can be identified, which should be considered as goals for research. One of these is to establish the bases for identifying the biological and clinical implications of the presence of potentially pathogenic microorganisms in the airways that should be more clearly elucidated according to the COPD phenotype. Another urgent area of research is the role of long-term preventive antibiotics. Clinical trials need to be carried out with inhaled antibiotic therapy to help clarify the profile of those antibiotics. The role of inhaled corticosteroids in patients with COPD and CBI needs to be studied to instruct the clinical management of these patients. Finally, it should be explored and confirmed whether a suitable antimicrobial treatment during exacerbations may contribute to breaking the vicious circle of CBI in COPD. The present review addresses the current state of the art in these areas to provide evidence which will enable us to progressively plan better healthcare for these patients.

## 1. Introduction

One of the situations with a major potentially negative impact on patients with chronic obstructive pulmonary disease (COPD) and which generates considerable uncertainty in clinical practice is the presence of a chronic bronchial infection (CBI). Generally, this clinical situation has been associated with the presence of bronchiectasis; however, there is accumulating evidence to indicate that COPD patients may have a CBI that causes a major clinical impact and which affects the progression of the disease without the presence of bronchiectasis being necessary [1]. CBI may have important clinical consequences in patients with COPD. Infected patients have an increased local and systemic inflammation, more frequent and severe exacerbations, and an accelerated progression of the disease [1]. Therefore, there is sufficient evidence to consider the COPD patient with CBI as a clinically relevant phenotype within the spectrum of COPD clinical presentation [2]. However, no management strategies have been developed and tested specifically for the treatment of CBI; instead, research has been conducted into treatments to prevent exacerbations in patients with CBI [3,4]. This clinical context represents a significant challenge for the clinician and generates numerous research questions that require a solution before the clinical questions posed may be answered.

In this context, it is necessary to reach a consensus that takes into account the available evidence and can serve both as support to the clinician who has to care for these patients and as the basis for identifying future lines of research. The aim of this document is therefore to review the available evidence in order to clarify the concepts and provide a basis for future clinical developments in the coming years. We will start by addressing a number of relevant but controversial issues.

## 2. Bronchial Colonization versus Bronchial Infection

Over the last few years, there has been an increasing body of evidence about the presence of a “normal” microbiota in the lower respiratory tract in healthy subjects [5]. The microorganisms that make up this microbiota are present in low concentrations and are usually uncultivable in conventional culture protocols used in routine clinical microbiology laboratories for the diagnosis of respiratory tract infections. However, these microorganisms have been identified in the respiratory secretions from the lower airways thanks to the introduction of metagenenomic approaches, including 16S rRNA amplification and whole genome sequencing analysis [5,6]. The most common species are anaerobic microorganisms, including *Prevotella* spp., *Veillonella* spp., and *Streptococcus* spp. [7].

Within this scenario, it is difficult to follow the standard definitions of infection or colonization. The former normally refers to tissue invasion, whereas the latter denotes the presence of the microorganism without exercising a clear pathogenic effect. In COPD, the bacteria grow over the mucous layer but rarely invade other compartments. Traditionally, the term “bronchial colonization” has been used to define the presence of potentially pathogenic microorganisms (PPM) in the lower respiratory tract of patients with chronic respiratory diseases such as cystic fibrosis (CF), bronchiectasis, or COPD during stable stages [8]. However, even though these microorganisms do not normally invade the tissues and are only present over the mucous layer, others prefer to use the term “infection”. Further, another suggested way of describing the situation is the concept of “pathogenic colonization”, which highlights the fact that, despite colonization, a pathogenic effect is produced [9]. Pathogenic colonization is associated with the increase in bacterial load, the direct bacterial interaction with the respiratory epithelium, the effect of bacterial exoproducts, and also the triggering of the immunology system and inflammatory cascade.

Nowadays, the term CBI has gained more adepts as a way of simplifying the model of alien microorganisms in the respiratory tract and to reinforce their clinical relevance and consequences. CBI is also used in bronchiectasis and CF patient management guidelines to make them more understandable [10,11]. It refers to an infection rather than a colonization or even a pathogenic colonization and is used when designing therapeutic measures in the management of COPD. A recent consensus document has suggested an operational definition of CBI for COPD as the growth of the same PPM in at least 3 sputum cultures in a year, separated by at least 1 month each [12].

The presence of the different microorganisms belonging to the normal microbiota and the equilibrium between them normally protects from the introduction of the so-called “PPM”, which might progress from the upper to the lower respiratory tract and consolidate their presence in the distal part of this niche. The group of PPM includes, among others, *Haemophilus influenzae, Streptococcus pneumoniae,* or *Pseudomonas aeruginosa*, the latter being a traditional indicator of worsening of the respiratory function [13] (Table 1). It has been demonstrated that patients with COPD have an altered microbiota with less abundance and a disbalance of the protective bacteria. Furthermore, this abnormal microbiota has been correlated with the overgrowth of well-known pathogens, including *P. aeruginosa* [14]. This altered situation has been termed dysbiosis, contrary to the situation in healthy populations, known as eubiosis. Compared with non-COPD affected individuals, an increase in the relative abundance of bacteria belonging to *Proteobacteria phylum* (particularly *Haemophilus* genus) and disappearance or relative decrease of that of *Bacteroidetes* phylum (especially *Prevotella*) and *Firmicutes* phylum (*Streptococcus* and *Peptoestreptococcus*) have been observed in COPD [6]. Nevertheless, other studies have shown that, during exacerbations, it is the balance of microorganisms that is affected rather than there being presence of new bacteria [15].

## 3. Exacerbations in the Context of Chronically Infected COPD Patients

COPD is characterized by recurrent episodes of exacerbations defined by an acute increase in respiratory symptoms [16]; however, not all exacerbations share the same characteristics. Recent studies have further characterized four different phenotypes of exacerbations: bacterial, viral, inflammatory (eosinophilic), and pauci-inflammatory, with more than 50% of them being bacterial or viral in nature [17]. Interestingly, the phenotype of the exacerbation remains constant in a given patient [18], and in the case of bacterial exacerbations, these episodes are usually characterized by increased dyspnea, increased sputum production, and changes in the color of sputum [19]. Repeated sputum sampling has demonstrated that the same microorganisms isolated during the exacerbation may persist after recovery in up to 50% of cases [20], which could be the cause of a residual CBI, especially when no appropriate eradicative antimicrobial treatment is provided [21]. In addition, the presence of CBI by the usual PPM is a risk factor for frequent and more severe bacterial exacerbations [22], thus establishing a vicious circle of CBI and bacterial exacerbations, modulated by the host’s defense mechanisms (which are usually impaired in patients with COPD) and the antibiotic treatment, either during exacerbations or in the stable phase.

**Figure 1:**
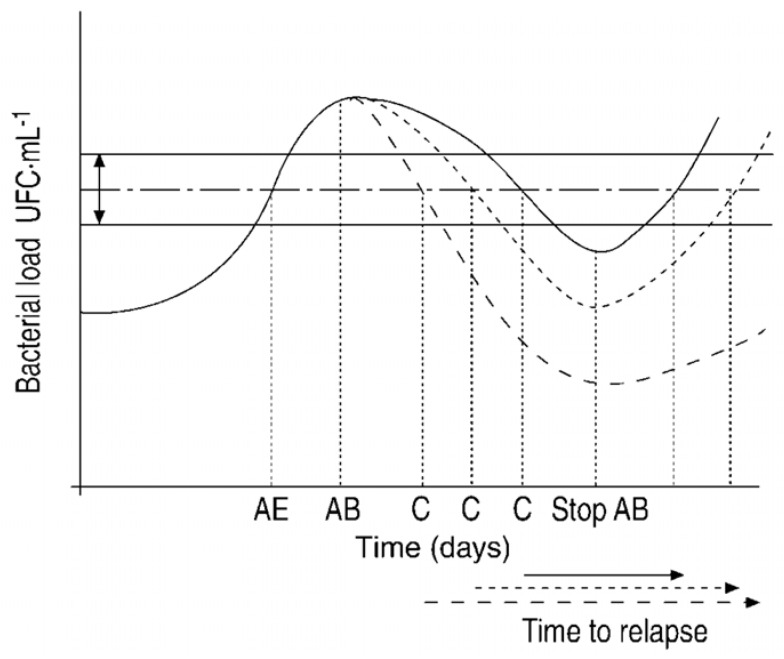
Areas of controversy about long-term antibiotic therapy in COPD. AE: Acute exacerbation, AB: antibiotic therapy (Continuous line: AB1; short dotted line: AB2; long dotted line: AB3); C: cure; Horizontal dotted/dashed line: clinical threshold within certain limits of variability according to modifying factors (horizontal continuous lines). Under AB therapy, the concentration of PPM decreases, and when the threshold is crossed again, the clinical symptoms disappear (cure). When the intensity and speed of the bactericidal activity of the AB is increased, recovery occurs more rapidly, and the time to the next exacerbation (horizontal arrow) is lengthened. AB activity produces a “fall” in bacterial concentrations, which, if not completely eradicated after the pressure of the antimicrobial agent is removed, “rise” again. Reproduced with permission of the © ERS 2020. European Respiratory Journal 20 (36 suppl) 9s–19s; DOI: 10.1183/09031936.02.00400302 Published 1 July 2002.

Three different mechanisms have been put forward to explain the pathogenesis of bacterial exacerbations. The first is the “Fall and Rise” hypothesis (Figure 1), which suggests a primary CBI as a result of impaired local defenses in smokers with COPD. The increase in bronchial bacterial load and associated increase in local and systemic inflammation in a patient with CBI will cause the symptoms of exacerbation [23]. The second theory postulates the change of strain in the PPM chronically infecting the airways as a mechanism to explain the occurrence of an exacerbation. In this case, the balance between the PPM present in the airway and the host would be disrupted by the new strain, against which no immune protection exists [24]. The third suggests that a viral infection may be the trigger that impairs the local defenses [25] and facilitates the superinfection by PPM. This has been demonstrated in human experimental models [26] and in naturally occurring exacerbations [27].

Whichever the mechanism, it is clear that a strong relationship exists between CBI and infective exacerbations (Table 2) and that this relationship justifies the use of more active bactericidal antibiotics to treat exacerbations of COPD with the objective of not only improving the symptoms but also eradicating as much bacteria as possible to avoid residual CBI and delay the recurrence of exacerbations [21,28]. Furthermore, this relationship also partly explains the efficacy of long-term antibiotic therapy in stable COPD to prevent recurrent bacterial exacerbations [29,30].

The phenotype of exacerbation is usually linked to a clinical phenotype of COPD. Most patients with CBI and frequent bacterial exacerbations are usually chronic producers of sputum that can be colored, even in stable state [31], usually have more respiratory symptoms and an impaired quality of life, and may have bronchiectasis when explored by a thorax CT scan. This is what constitutes the so-called infective phenotype of COPD [1]. This infective phenotype, characterized by the vicious circle of chronic and acute bronchial bacterial infection and the presence of bronchiectasis, is supported by a recent study that observed the development or appearance of new bronchiectasis in patients with COPD who presented the characteristics of this phenotype: presence of muco-purulent or purulent sputum, admission to hospital due to exacerbations, and the isolation of potentially pathogenic microorganisms from the sputum [32].

Therefore, more than half of exacerbations of COPD have a bacterial etiology, and impaired bronchial defense mechanisms in smokers with COPD and an inadequate or lack of antibiotic treatment may result in persistence of the bacteria or CBI. On the other hand, the presence of CBI is a risk factor for frequent and more severe exacerbations, which constitute a vicious circle, with negative consequences for the patient such as more respiratory symptoms, impaired quality of life, development of bronchiectasis and progression of the disease. Suitable antimicrobial treatment during exacerbations and in some cases also in the stable phase may contribute to breaking this vicious circle.

## 4. *P. aeruginosa* in Patients with COPD

*P. aeruginosa* is one of the most virulent opportunistic respiratory bacteria, for several reasons: (1) It forms biofilms that prevent the action of antibiotics; (2) it frequently develops antimicrobial resistance; (3) it persists in the bronchial mucosa, and (4) it is normally associated with poor outcomes in chronic airway diseases [33]. Once CBI by *P. aeruginosa* is established, it is rarely eradicated [34], and as a consequence, international guidelines in both diseases strongly recommend an aggressive antibiotic treatment to eradicate it the moment *P. aeruginosa* is first isolated in respiratory samples, in an attempt to avoid the progression to a CBI [10,11,35,36,37].

Out of an exacerbation episode, cross-sectional studies have shown that *P. aeruginosa* accounts for 4–15% of all PPM able to induce CBI [1,37,38]. Multiple risk factors have been identified with the acquisition of a *P. aeruginosa* infection in COPD: previous isolation of *P. aeruginosa*, multiple courses of systemic antibiotics or steroids, more advanced disease, bronchiectasis, current smoking habit, and a previous stay in an intensive care unit [1,37,38,39,40,41,42]. However, the relationship between the isolation of *P. aeruginosa* and poor outcomes in patients with COPD is more controversial. Jacobs et al. prospectively studied 181 COPD patients, 40% of whom had *P. aeruginosa* isolation. Both the first isolation and multiple isolations of *P. aeruginosa* were linked to higher mortality [43]. Conversely, Boutou et al. [44] concluded that a single isolation of *P. aeruginosa* is not associated with higher mortality in COPD patients. Regarding exacerbations, Rosell et al. [45], pooling the results of six studies that obtained the microbiological sample through the protected specimen brush, observed that *P. aeruginosa* was associated with a greater number and severity of exacerbations, regardless of the bacterial load. However, Murphy et al. [46] in a 10-year prospective study concluded that only the acquisition of a new strain (but not all positive cultures) of *P. aeruginosa* was associated with an increased incidence of exacerbations. Recently, Eklöf et al. [13] performed an epidemiological study in 22,053 COPD outpatients, 4.2% of whom had at least one positive culture by *P. aeruginosa*, concluding that *P. aeruginosa* strongly and independently predicted an increased risk of hospitalization and all-cause death. However, some of the studies that analyzed the exacerbations and mortality related to *P. aeruginosa* had obtained the microbiological samples during an exacerbation period [47,48,49,50,51,52,53].

One of the most interesting controversies is whether *P. aeruginosa* is a marker of disease severity or the cause of exacerbations and rapid deterioration of COPD patients. Although there is still not a clear answer to this question, Martinez-Solano et al. [51] provided some evidence supporting the latter hypothesis by observing patterns of *P. aeruginosa* infection and evolution in COPD that resembled those observed in CF. Again, the lack of agreement in this area can be seen in the study by Rakhimova et al. [54], who concluded that *P. aeruginosa* as found in COPD has a frequent turnover of different clones of *P. aeruginosa,* which differ from those observed in CF, in which it is usually seen as a chronic carriage of the same *P. aeruginosa,* and the mucoid form is more frequent.

A better understanding of the influence of *P. aeruginosa* infection on COPD morbidity and morbidity in outpatients and the experience gained from treating bronchiectasis and CF would help us to implement specific therapies and new procedures for the prevention, diagnosis, and treatment of *P. aeruginosa* infection in COPD patients. 

## 5. Macrolides in Chronically Infected COPD Patients

Macrolides are the most widely used class of antimicrobials for long-term administration to prevent exacerbations in patients with COPD. The use of macrolides may be effective because, although most PPM are usually resistant to macrolides, azithromycin, in addition to its antimicrobial activity, protects the bronchial epithelium during infections and may interfere in the biofilm, making the PPM more sensitive to other antibiotics such as quinolones [55]. It is of note that the use of macrolides has been associated with a wide variety of immunomodulatory and anti-inflammatory effects. Macrolides reduce mucus secretion and decrease total cell counts, neutrophil chemotaxis, and levels of inflammatory markers in sputum. Furthermore, macrolides have inhibitory effects on bacterial virulence and on the formation of biofilms and also exert inhibitory effects on viral infections [56].

Although the long-term use of macrolides is recommended for the prevention of exacerbations in bronchiectasis [35], it is well documented that a significant proportion of COPD patients with frequent exacerbations, especially those with CBI, may also have bronchiectasis [2]. Similarly, studies that demonstrate the reduction of exacerbations with macrolides in bronchiectasis include a significant number of patients with coexistent COPD [57]. However, some trials of macrolides in COPD specifically excluded patients with bronchiectasis demonstrated by CT scans and, nonetheless, also provided highly significant results in terms of reduction of exacerbations [58]. The efficacy of macrolides in prevention of exacerbations in COPD may therefore be linked to the presence of bronchiectasis in some patients, but it may also be determined by the presence of recurrent infective exacerbations and CBI or perhaps their anti-inflammatory activity [59,60].

The recent meta-analysis of the efficacy of macrolides included in the European Respiratory Society (ERS)/American Thoracic Society (ATS) guidelines on the prevention of exacerbations in COPD included placebo-controlled randomized trials lasting at least one year [61]. The macrolide regimens used included erythromycin 200 to 400 mg daily [62], erythromycin 250 mg twice daily [63], azithromycin 250 mg daily [64], and azithromycin 500 mg three times per week [58]. Macrolide therapy decreased the rate of COPD exacerbations (rate ratio 0.76, 95% CI 0.68 to 0.86), and increased the time to the first exacerbation (mean difference 81.5 more days, 95% CI 53.3 more to 109.8 more). Macrolide therapy also reduced the proportion of patients who developed an exacerbation (57% versus 68%, risk ratio 0.84, 95% CI 0.76 to 0.92) [61]. A summary of the trials on macrolide therapy in COPD is presented in Table 3.

The two regimens with the most supporting evidence are azithromycin 500 mg/day, three days/week or azithromycin 250 mg/day, every day, the former being the most widely extended. Continuation of the treatment should be based on the clinical response (reduction of exacerbations) and the appearance of any side effects.

The meta-analysis concluded that there was no evidence that macrolide therapy increased serious adverse events collectively, although there was an increased incidence of a hearing decrement measured by audiometry. The effects of macrolide therapy on the acquisition of macrolide-resistance were uncertain [61], although given the high prevalence of COPD, it is a current concern whether this may contribute to macrolide resistance in the community. Finally, the risk of ventricular arrhythmias after the use of macrolides has to be considered, particularly when there are possible interactions with other medications or in patients with significant cardiac comorbidity [61,65,66].

The ERS/ATS guidelines concluded with a conditional recommendation in favor of the use of macrolides to prevent exacerbations in patients with moderate to very severe COPD and exacerbations despite optimal inhaled therapy [61]. Similarly, the Spanish COPD guidelines recommend the use of long-term macrolides in patients with COPD and at least 3 exacerbations in the previous year while on optimal inhaled therapy [67]. This last document includes the recommendation to prescribe this therapy only in reference centers with a close follow-up of the possible side effects, such as hearing loss, liver and cardiac complications, and the monitoring of microorganisms in sputum cultures with analysis of their susceptibility to antibiotics.

## 6. Inhaled Corticosteroids in Chronically Infected COPD Patients

It has been consistently described that inhaled corticosteroids (ICS) reduce exacerbations and improve symptoms and quality of life for patients with advanced COPD [71]. Accordingly, ICS are part of the therapeutic strategy for COPD patients [67,72]. In addition, there is increasing recognition that ICS may impair the critical components of the immune system that are essential for effective host-defense against respiratory pathogens in COPD patients, resulting in an increased risk of respiratory infections [73]. As mentioned above, the available data indicate that the lower respiratory tract is colonized by complex bacterial microbiota in which dysbiosis alterations may be present in COPD [15]. ICS use in patients with COPD is associated with adverse effects: altering the antiviral immune response [74], modifying the composition of the microbiome [75], increasing bacterial load [76], the risk of respiratory infections, pneumonia [77], and mycobacteria infection [78]. Accordingly, ICS could theoretically promote “de novo” respiratory infections and pneumonic episodes either by inducing the growth of bacteria within the existing lung microbiota or by contributing to the acquisition of new bacteria from the environment [79]. Additionally, around 50% of ICS-induced pneumonias in COPD occur following a viral illness [80].

In this scenario, the impact of an ICS in patients with COPD and an already existing CBI must be carefully evaluated. This has led to a shifting paradigm, with increasing recognition that the potentially detrimental effects of ICS on anti-microbial immunity should be carefully weighed up against any potentially beneficial anti-inflammatory and clinical effects [79]. Consistently, current guidance documents advocate more selective ICS use in COPD than in asthma [67,72]. Accordingly, some authors have argued that the therapeutic response to ICS could be considered a pivotal aspect of a therapeutic strategy in a patient-centered approach [81]. Here, the role of blood eosinophils has been proposed as a potential ICS-response marker [82]. Although its role in the context of CBI has not been sufficiently explored, it should be considered a marker worth exploring in the future.

In a recent post-hoc long-term observational study of a historical cohort of 201 COPD patients who were carefully characterized, including airway microbiology, and followed for a median of 84 months, less than 100 circulating eosinophils/µL, together with the presence of CBI, increased the risk of pneumonia in COPD patients treated with ICS [41]. Therefore, the use of ICS must outweigh the potential increase in the risk of infective complications in these patients [35]. In cases where ICS are deemed necessary, it seems reasonable to advocate using them at the lowest dose possible [83]. Thereafter, the risk/benefit ratio of their use should be re-evaluated in patients who do not have high blood eosinophil levels or traits consistent with concomitant asthma and avoided when necessary [67]. Additionally, the maintenance of ICS should be re-assessed if there is a lack of response or infective complications appear.

## 7. Long-Term Systemic or Inhaled Antibiotic Treatment

**Figure 2:**
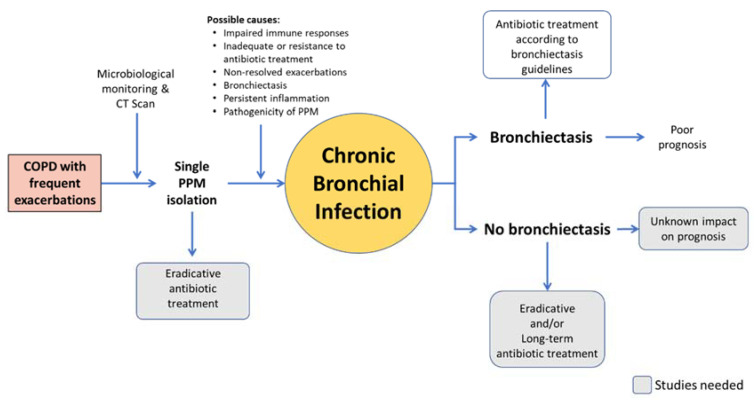
Areas of controversy about long-term antibiotic therapy in COPD.

Long-term antibiotic therapy seems a reasonable option to eradicate or reduce the bacterial load of these patients’ airways, thereby reducing its negative impact, given either systemically or inhaled (Figure 2).

### 7.1. Systemic Antibiotics Other than Macrolides

In patients with bronchiectasis, long-term systemic antibiotic therapy is associated with various clinical benefits [84], while in patients with stable COPD, the evidence is scarcer and based largely on studies with macrolides [85,86,87]. The first publications on the use of non-macrolide antibiotics in stable COPD patients were published more than 60 years ago [29,88,89,90,91,92,93,94,95,96,97]. More recent ones are summarized in Table 4.

In all these trials, there was a great variability in the design, many of which were small and of short duration. In most of the older studies, the antibiotics tested are not currently used as a first-line treatment in respiratory infections, such as tetracyclines and sulphamide drugs. In addition, hardly any studies considered repeated isolation of PPM as an inclusion criterion, mainly focusing on patients with chronic bronchitis and a history of exacerbations. Despite this and although some results were discordant, several clinical benefits were observed, including reductions in the amount or purulence of the sputum, exacerbations, days off work and bacterial load (even eradication), and an improvement in quality of life [29]. Only one study, which was retrospective, included patients based on a microbiologic criterion, demonstrating that fluoroquinolones were associated with an increased eradication rate compared to macrolides [85]. The adverse effects reported were generally scarce and mild in most cases. Bacterial resistance was barely investigated in the oldest publications, and in the most recent ones, a worrying development of resistant microorganisms was observed in only one.

### 7.2. Inhaled Antibiotics

In recent years, inhaled antibiotic therapy has experienced a notable increase, mainly based on the good results in the treatment of IBC in CF [98] and in non-CF bronchiectasis [10,35,36,99]. Inhaled antibiotics have shown to reach a high concentration in the bronchial tree, decreasing the bacterial load in the airways and favoring clinical improvement, with few systemic adverse effects. However, clinical trials demonstrating its efficacy in COPD patients are lacking. Only some studies have evaluated the efficacy of inhaled colistin, tobramycin, or amoxicillin-clavulanic acid in patients with COPD (Table 5) [100,101,102,103]. These were either single-center uncontrolled trials or retrospective studies. Although they contain slight variations due to methodological differences, they showed that inhaled antibiotic use in COPD patients with CBI was associated with a reduction in inflammatory parameters, bacterial load (even eventual eradication), exacerbation rate, and hospital visits. Adverse effects were not frequent, nor was the emergence of resistant microorganisms. In addition, there are several ongoing clinical trials on the effect of ciprofloxacin, levofloxacin, and amikacin in patients with COPD and CBI.

The lack of scientific evidence prevents COPD treatment guidelines from establishing recommendations on its use [67,72]. Nevertheless, many clinicians prescribe these treatments in COPD patients with CBI (mainly due to *P. aeruginosa*), with poor clinical progression despite adequate treatment, including failure after long-term macrolides. In the absence of more information, the dosage and precautions at the time of administration should be the same as in bronchiectasis (Table 6). Notably, the choice of the antibiotic does not depend on the antibiogram, since inhaled antibiotics reach bronchial mucosa concentrations well above the minimum inhibitory concentration [104].

Consequently, clinical trials should be carried out to establish the utility of long-term antibiotic therapy in COPD and resolve the many existing issues. Some of the controversies regarding these trials include: (1) Patients with bronchiectasis should probably not be excluded from these trials since they are present in a large number of patients with severe COPD and CBI. (2) It is necessary to define what type of patients should be candidates for long-term antibiotic therapy and using which modality (oral vs. inhaled). This may depend on the PPM responsible (for example, inhaled antibiotics for PA and oral treatment for *H. influenzae*) but also on the tolerance to each of the administration routes, the patient’s respiratory function, etc. (3) It must be decided whether to treat the primary infection by *P. aeruginosa* with inhaled antibiotics, which are the best drug and best treatment regimen (continuous treatment vs. on-off cycles), what the optimal duration of treatment is, etc.

## 8. Future Directions

While reviewing the current literature, several challenges can be identified that should be considered as goals for research: (1) The bases for identifying the biological and clinical implications of the presence of PPM in the airway should be established; (2) the clinical implications of the presence of PPM in COPD patients should be more clearly elucidated according to the underlying clinical COPD phenotype; (3) the role of long-term preventive antibiotics in relation to the type of underlying inflammation should be investigated further; (4) clinical trials should be conducted with inhaled antibiotics in patients with COPD to help to clarify which antibiotics show greater efficacy and safety in these patients; (5) the role of ICS in the treatment of patients with COPD and CBI needs to be addressed urgently in order to guide the clinical management of these patients on a day-to-day basis; (6) finally, it should be explored and confirmed whether a suitable antimicrobial treatment during exacerbations, and in some cases also in the stable phase, may contribute to breaking the CBI vicious circle in COPD. Additionally, different biomarkers or biological pathways are looming in the future as a potential target inflammatory mechanism. There is an increasing body of findings highlighting the beneficial role of omega 3 precursors and metabolites in treating several respiratory conditions that show an advanced airway inflammation and hyperresponsiveness [105,106,107]. Very recently, some guidelines on how to manage the CBI in stable COPD have started to emerge [12] as a response to an urgent need to unify the knowledge acquired so far and provide evidence which will enable us to plan progressively better healthcare for these challenging patients.

## Figures and Tables

**Table 1:** List of microorganisms potentially implicated in chronic obstructive pulmonary disease (COPD).

Potentially Pathogenic Microorganisms	Non-Potentially Pathogenic Microorganisms
*Haemophilus influenzae* *Streptococcus pneumoniae* *Moraxella catarrhalis* *Pseudomonas aeruginosa* Other non-fermenting Gram-negative bacilli: ○ Achromobacter xylosoxidans ○ Acinetobacter baumannii ○ Alcaligenes faecalis ○ Stenotrophomonas maltophilia ○ *Pseudomonas* spp. *Klebsiella pneumoniae* Other *Enterobacterales* ○ Escherichia coli ○ Klebsiella aerogenes ○ Enterobacter cloacae ○ Serratia marcescens ○ *Proteus* spp. ○ *Providencia* spp. ○ *Citrobacter* spp. *Staphylococcus. aureus*, including methicillin-resistant isolates *Pasteurella multocida*	Viridans group streptococci *Gemella morbillorum* Commensal neisseria species *Staphylococcus epidermidis* and other coagulase negative staphylococci *Micrococcus* spp. *Enterococcus* spp.

**Table 2:** Relationship between chronic bronchial infection (CBI) and bacterial exacerbations of COPD.

Clinical impact of CBI in COPD
Patients with CBI suffer from more frequent exacerbations.
Patients with CBI suffer from more severe exacerbations.
Incomplete eradication after antibiotic treatment of exacerbations may result in CBI.
PPM identified during CBI are the same ones that produce exacerbations in most cases.
Both CBI and bacterial exacerbations may be identified by the production of muco-purulent or purulent sputum.
Long-term antibiotic treatment of CBI can reduce the frequency of exacerbations.
The presence of CBI and hospital admissions for exacerbations are associated with the development of bronchiectasis in COPD.

**Table 3:** Clinical trials of long-term macrolide treatment for COPD.

Study	Design and Treatment Groups	Patient Population	Results
Suzuki et al, 2001 [62]	Randomized, non-blinded study of 109 patients, 55 treated with erythromycin 200–400 mg/day and 54 in the control group for 1 year	Mean age 70 years, mean FEV1 between 1.3 to 1.47 L	Reduction in common colds and exacerbations in the antibiotic group.
Seemungal et al, 2008 [63]	Randomized, double-blind, placebo-controlled study. 109 patients, 53 treated with erythromycin 250 b.i.d and 56 with placebo for 1 year	Mean age 67.2 years, mean FEV1(%) = 50%	35% reduction of exacerbation frequency with antibiotic (*p* = 0.006). Median time to first exacerbation was 271 vs. 89 days in the placebo arm (*p* = 0.02). Reduction in duration of the exacerbation with macrolide.
Pomares et al, 2011 [68]	Retrospective study of 24 COPD patients treated with azithromycin 500 mg three times per week for 1 year	Mean age 70.9 years, mean FEV1(%) = 32.2%, a mean of 3.3 hospitalization and 7 exacerbations the previous year	A 58.9% reduction in exacerbations and 61.2% reduction in hospitalizations compared with previous year without macrolides.
He et al, 2010 [69]	Randomized, double-blind, placebo-controlled study of 36 patients, 18 treated with erythromycin 125 mg t.i.d and 18 with placebo for 6 months.	Mean age 69 years. Mean FEV1(%) = 43%	Reduction in total numbers of sputum cells and neutrophil elastase. A reduction of 44% in relative risk of exacerbation with the antibiotic. Delayed time to first exacerbation with macrolide.
Blasi et al, 2010 [70]	Open label, randomized, uncontrolled trial of 22 patients with COPD and tracheostomy, 11 treated with azithromycin 500 mg 3 days a week for 6 months and 11 in standard care group	Mean age 72 and 73 years. No lung function available. 91% and 73% were colonized.	Longer time to the first exacerbation with the macrolide. Estimated hazard ratio for first exacerbation associated with standard care 5.41 (95% CI: 1.67–17.5). Reduction in hospitalization with azithromycin.
Albert et al, 2011 [64]	Randomized, double-blind, placebo-controlled trial of 1142 patients. 570 assigned to azithromycin 250 mg daily and 572 to placebo for a year.	Mean age 66 years. Mean FEV1(%) 39-40%, up to 50% required hospital visit for exacerbation the previous year.	Reduction in risk of exacerbation with azithromycin (*p* <0.001). Median time to first exacerbation prolonged from 174 with placebo to 266 with macrolide. Hazard ratio for time to the first exacerbation was 0.71 (95% CI: 0.61 to 0.83; *p* <0.001)
Uzun et al., 2014 [58]	Randomized, double-blind, placebo-controlled study of 92 patients, 47 assigned to azithromycin 500 mg 3 times a week for 12 months and 45 to placebo	Mean age 65 years, mean FEV1(%) = 45%, at least 3 exacerbations the previous year. Patients with bronchiectasis in CT were excluded.	Reduction of exacerbation of 42% with azithromycin (reduction risk 0.58, 95% CI: 0.42–0.79; *p* = 0.001). Median time to first exacerbation was 59 days with placebo and 130 with azithromycin (*p* = 0.001).
Vermeersch et al., 2019 [30]	Randomized, double-blind, placebo-controlled trial of 301 patients admitted for an exacerbation of COPD. 147 assigned to azithromycin 500 mg 3 for days and 250 mg every 2 days for 3 months and 154 assigned to placebo	Mean age 67 years, mean FEV1(%) = 37%, at least 1 exacerbation the previous year.	There was no change in the treatment success. Azithromycin decreased treatment failure: 49% azithromycin and 60% placebo (HR 0.73; 95% CI: 0.53–1.01; *p* = 0.052). Clinical benefits were lost 6 months after withdrawal.

FEV1: forced expiratory volume in 1 s; bid: bis in die; COPD: chronic obstructive pulmonary disease; tid: ter in die.

**Table 4:** Main publications on the long-term use of systemic antibiotics in patients with stable COPD.

Study and Design	Study Population	Main Results	Other Results
**Sethi et al. (2010)** [29] Double-blind, randomized, placebo-controlled trial Moxifloxacin 400 mg once daily for 5 days, every 8 weeks for a total of six courses vs placebo 1157 patients 48-week treatment period Further 24-week follow-up	Stable COPD patients with chronic bronchitis and at least two exacerbations in the 12 months prior to enrolment. Exclusions: tendon disease, arrhythmias, hepatic impairment, other respiratory disease, chronic colonization of pathogenic organisms resistant to moxifloxacin, systemic or inhaled antibiotic therapy during the 6 weeks prior to screening, need for home ventilatory support for COPD.	Reduced odds of exacerbation: 20% in the ITT population 25% among the PP population 45% in PP patients with purulent/mucopurulent sputum at baseline.	No differences in hospitalization or mortality rates, lung function or changes in SGRQ total scores. Significant difference in favor of moxifloxacin in the SGRQ symptom domain. More adverse events with moxifloxacin (mainly gastrointestinal). Moxifloxacin treatment was not associated with consistent changes in moxifloxacin susceptibility.
**Brill et al. (2015)** [84] Single-blind, randomized, placebo-controlled trial 4 treatment groups -Moxifloxacin 400 mg daily for 5 days every 4 weeks -Doxycycline 100 mg/day -Azithromycin 250 mg 3 times a week - Placebo 99 patients 13-weeks treatment period	Stable COPD patients with chronic bronchitis. Exclusions: Other clinically significant respiratory disease, COPD exacerbation in the 4 weeks preceding screening or before randomization, hepatic or renal impairment, evidence of tuberculosis, uncontrolled hypertension, prolonged Q-T interval, long term antibiotics for any reason.	Non-significant reduction in bacterial load in the treatment arms, compared to placebo. No significant improvements in bacterial load measured by 16S qPCR or in airway inflammation.	More treatment-related adverse events with moxifloxacin. Mean inhibitory concentrations increased x3 times over placebo in all treatment arms.
**Pettigrew (2016)** [85] Retrospective study Fluorquinolones vs. macrolides 77 patients 15 years follow-up	COPD patients with chronic bronchitis and at least 1 *H. influenzae* isolation. Exclusions: asthma, bronchiectasis, inability to comply with a schedule of monthly clinical visits, immunosuppressive or other life-threatening disorders.	Fluoroquinolone administration was associated with increased *H. influenzae* eradication compared to macrolides. No difference in *H. influenzae* eradication when comparing macrolide administration to no antibiotic.	19% strains developed x4 increase in azithromycin MIC. No mutations in quinolone resistance-determining regions.

ITT: intention to treat; PP per protocol; SGRQ: St. George’s Respiratory Questionnaire; COPD: chronic obstructive pulmonary disease; MIC: Minimum inhibitory concentration.

**Table 5:** Main publications on the use of inhaled antibiotics in patients with stable COPD.

Study Type	Study Population	Antibiotic Studied and Doses	*n*/Treatment Duration	Main Results	Other Results	Antibiotic Resistance
**Dal Negro (2008)** [100] Single arm prospective intervention study	Severe COPD patients chronically colonized with *P. aeruginosa* resistant to oral/intravenous specific antibiotics. Exclusions: asthma; bronchiectasis; pregnancy or lactation; pneumonia; lung malignancy; immunosuppression; liver or renal insufficiency; cardiac failure; use of antibiotics in the previous 4 weeks; other infections.	Tobramycin Nebulizer Solution (TNS) TNS 300 mg/12 h	13 patients, 14 days further 6 months follow-up	TNS induced a significant reduction in sputum IL-1β, IL-8, ECP concentrations, and eosinophil count. At 6 months, *P. aeruginosa* eradication in two patients and density reduction in four. The incidence of exacerbations decreased by 42%.	TNS was well tolerated. Episodes of bronchospasm or other adverse effects were not reported. All patients completed the treatment with 100% compliance.	Atypical organisms were not detected during the study period in any of the patients. Respiratory viruses detected in the same period were influenza A/B (6), parainfluenza 1/2/3 (4), and RSV (2).
**Nijdam (2016)** [101] Two phase 1 single-arm prospective intervention studies	COPD patients, able to produce sputum. STONAC 1: stable COPD outpatients; STONAC 2: patients hospitalized for exacerbation. Exclusions: allergy to penicillin, amoxicillin or clavulanic acid, current pneumonia, and FEV1 post bronchodilator < 1.2 L (STONAC 1 only), systemic use of amoxicillin (STONAC 2 only).	Amoxicillin/clavulanic acid 1000 mg/200 mg powder for solution for injection (registered for intravenous administration) STONAC 1: ascending doses, up to 300:60 mg of amoxicillin-clavulanic acid. STONAC 2: amoxicillin clavulanic acid 200:40 mg, twice daily during hospitalization (with a maximum of 7 days).	STONAC 1: 8 patients. Each patient received 4 doses with at least 7 days between each dose. STONAC 2: 8 patients.	No clinically relevant deteriorations in FEV_1_ were observed. Minor side effects were reported in 30% of patients (cough, shortness of breath, bitter taste). One patient in STONAC 2 presented bronchospasm.	All plasma levels showed amoxicillin concentrations < 1.0 mg/L, indicating that systemic exposure is low. In both studies, sputum amoxicillin levels resulted well above MIC90.	Not available
**Bruguera (2017)** [102] Retrospective study	Severe COPD patients (FEV_1_ ≤ 50%) with chronic or intermittent colonization by *P. aeruginosa* who initiated treatment with nebulized colistin between 2010 and 2014. Exclusions: asthma, malignancy, unstable heart disease, main diagnosis of bronchiectasis.	Colistimethate sodium Colistimethate sodium 1 million IU/12 h administered through the I-neb adaptive aerosol delivery device.	36 patients, 5-year review. Comparison between the year prior to and the year after starting the treatment.	Significant reduction in the number and length of hospitalizations. No reduction in the number of ambulatory exacerbations. Eradication of *P. Aeruginosa* in 38.9% of patients.	The effects of treatment did not depend on COPD severity, or on the presence/extension of bronchiectasis.	Resistance to colistin only in bacteria known as constitutively resistant.
**Montón (2019)** [103] Retrospective study	Severe COPD patients with chronic bronchial infection by *P. aeruginosa* who initiated a treatment with nebulized colistin between 2005 and 2015, in combination with long-term oral azithromycin. Exclusions: main diagnosis of bronchiectasis.	Colistimethate sodium + azithromycin -Colistimethate sodium with jet nebulizer (1–2 million IU/12 h) OR with I-neb adaptive aerosol delivery device (0.5–1 million IU/12 h). -Azithromycin 500 mg three times/week.	53 patients (32 in final analysis)10-year review. Comparison between the two years prior to and the two years after starting the treatment.	Significant reduction in the number of exacerbations (38.3%). Decrease in exacerbations due to *P. aeruginosa* (from 59.5% to 24.6%). *P. aeruginosa* eradication rate of 47% and 28% at 12 and 24 months.	Intolerance due to respiratory symptoms in 11 (20.8%) of the initial 53 patients.	No increase in percentage of multi-resistant *P. aeruginosa*. No resistance to colistin was observed during follow-up.

ITT: intention to treat; ECP: eosinophilic cationic protein; COPD: chronic obstructive pulmonary disease; STONAC: Safety and Tolerability of Nebulized Amoxicillin-Clavulanic Acid in Patients with COPD; FEV1: Forced expiratory volume in 1 s.

**Table 6:** Inhaled antibiotics marketed for administration by inhalation.

	Dose, Posology	Administration Time	Inhalation System
Aztreonam lysine, solution for nebulization	75 mg, 3 times a day, on/off *	2–3 min	e-Flow® (Altera)
Colistimethate, dry powder for inhalation	1,662,500 de UI, twice a day, continuous treatment	1–2 min	Turbospin®
Colistimethate, solution for nebulization	1–2 million IU, twice a day, continuous	Variable, depending on nebulizer	e-Flow®, Pari LC plus®
	0.5–1 million IU, twice a day, continuous	3–6 min	I-neb AAD®
Tobramycin, dry powder for inhalation	112 mg, twice a day, on/off *	~ 6 min	T-326 Inhalator
Tobramycin, solution for nebulization	300 mg/5 mL, twice a day, on/off *	Variable, depending on nebulizer	e-Flow®, Pari LC plus®
	300 mg/4 mL, twice a day, on/off *	Variable, depending on nebulizer	

* 28-day alternate cycles with and without treatment.
